# Biomarkers Are Consistent With Patient‐Reported Allergic Sensitization in Topical Steroid Withdrawal

**DOI:** 10.1111/all.70068

**Published:** 2025-09-25

**Authors:** Aditi Vijendra, Nadia Shobnam, Jalin Jordan, Pranav Thota, Ian A. Myles

**Affiliations:** ^1^ Epithelial Therapeutics Unit, National Institute of Allergy and Infectious Disease, National Institutes of Health Bethesda Maryland USA

**Keywords:** allergy, eczema, IgE, sensitization, topical steroid withdrawal

To the Editor,

Topical corticosteroids (TCS) are recommended by many international guidelines as first‐line therapies for atopic dermatitis (AD) and other inflammatory skin conditions [1]. However, mounting cases of systemic adverse reactions upon cessation of long‐term TCS use have been described as topical steroid withdrawal (TSW) [2]. We recently reported that reanalysis of a survey of patients with eczematous skin disease suggests TSW symptoms are distinguishable from AD using preliminary diagnostic criteria [3]. Further mechanistic insights suggest TSW induces hyperactivity in mitochondrial complex I, characterized by overproduction of NAD+ [3]. Several patients with TSW reported new sensitivities to previously tolerated exposures ranging from foods to skin care ingredients [4]. However, given that sensitivities can represent irritant reactions, further studies were needed to assess whether the specific sensitivities reported by patients with TSW were supported by IgE biomarkers.

After written, informed consent was obtained, serum samples were obtained from patients with TSW (*n* = 16), patients with AD who did not meet diagnostic criteria for TSW (*n* = 10), and healthy volunteers (*n* = 11). Samples were evaluated using the ALEX2 Allergy Xplorer, an ELISA‐based in vitro multiplex allergy test. Two samples from the TSW cohort failed instrument quality control and were excluded. Measurements included total and specific immunoglobulin E (IgE) reactivity to over 295 allergens, including 117 allergen extracts and 178 molecular allergens, associated with over 60 unique molecular allergens.

Allergen testing revealed no overall difference in specific IgE reactivity between groups (Figure 1A, Figure S1A,B), although patients with TSW displayed greater specific IgE to certain allergens than patients with AD (Figure 1B). Lasso regression identified reactivity to dust mite (Der f1, Der p2, Def p23, and Der f2), pet dander (dog; Can f1 and cat; Fel d1), and fungus (*Alternaria*; Alt a1 and *Aspergillus*; Asp f6) to be most predictive of TSW versus AD (Figure 1C). A greater proportion of patients in the TSW group were positive for at least one epitope within the same aeroallergen categories (Figure 2A). Patients with TSW displayed increased IgE to European house dust mites (*p* < 0.0001), dogs (*p* = 0.001), American house dust mites (*p* = 0.01), and the AD‐associated commensal yeast *Malassezia sympodialis* [5] (*p* = 0.02) (Figure S2A). A post hoc comparison suggested sensitization to dust mites diminished with >40 months of TCS avoidance (Figure S2B), suggesting reversibility. Overall sensitizations were consistent with clinical histories; however, they were only significant for seafood, grains, and trees (Figure 2B–L). Der p reactive IgE levels, while predictive of TSW versus AD, were reduced in those with symptom onset subsequent to TSW (Figure 2E). This may represent translocation of the IgE to the skin or other unexplored mechanisms.

**FIGURE 1:**
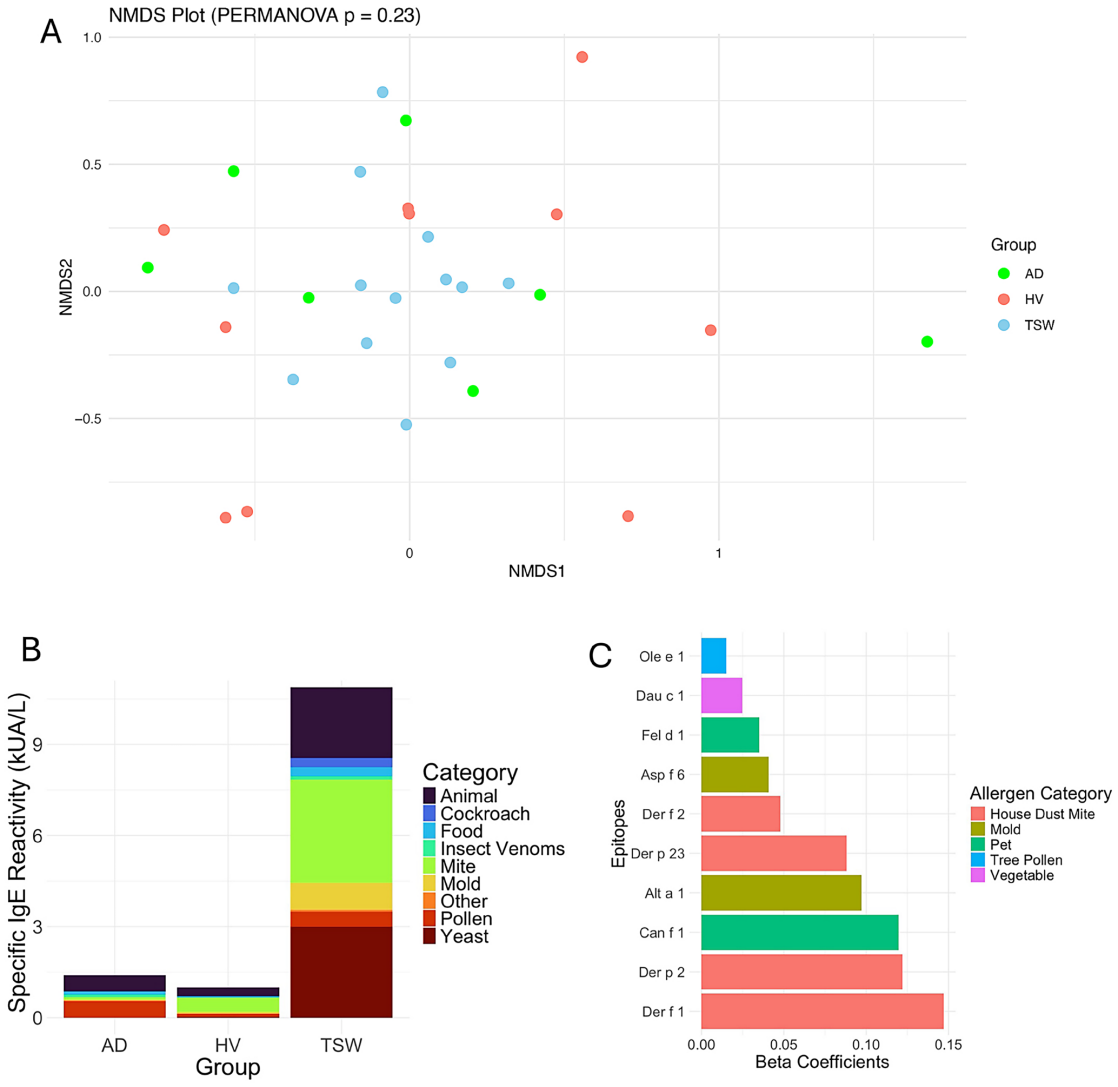
Serum samples from patients with TSW display greater sensitization to certain broad categories of allergens compared with patients with atopic dermatitis (AD) and health volunteers (HV). (A) Non‐metric MultiDimensional Scaling (NMDS) plot of overall similarity. (B) Mean specific IgE (kUA/L) by allergen category between groups. (C) Beta coefficients from lasso regression identifying most predictive allergen epitopes of TSW versus AD. The optimal lambda for the lasso was performed using glmnet with alpha of 1‐ and 10‐fold; then reanalyzed using the best lambda on scaled data.

**FIGURE 2:**
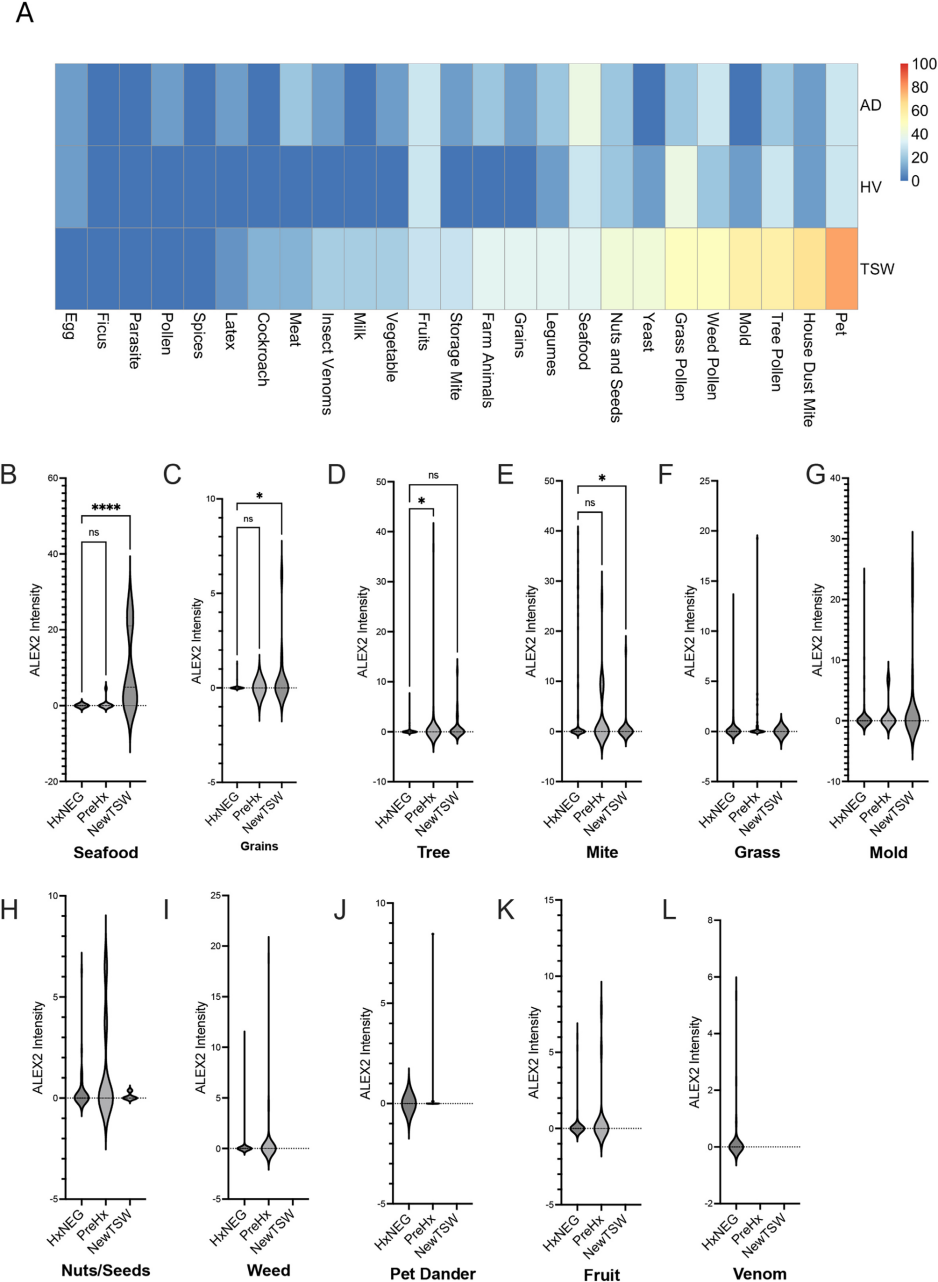
Sensitization to certain aeroallergens were identified as predictive of TSW versus AD. (A) Percent of patients with greater than 0.5 kUA/L of specific IgE for at least one epitope in an allergen category by group. (B‐L) Specific IgE reactivity (kUA/L) to several allergen categories between patients with no reported allergy, allergic history predating TSW onset, and new symptomatic onset subsequent to TSW symptoms. Antigens were groups by their subcategory. *p < 0.05, ****p < 0.0001, ns = not significant as calculated by ANOVA.

Increased sensitization to dust mites and pet dander among patients with TSW warrants further investigation among the environmental factors which may exacerbate or predispose patients to TSW. Furthermore, these data raise the possible relevance of *Malassezia sympodialis*, which has been linked to AD through several reported mechanisms [5]. Decreased abundance of the *Malassezia* genus was also reported in skin swabs from patients with TSW^3^, which could be interpreted as indicating an immune response to the fungi but could mean that the skin of patients with TSW is not conducive to the survival of *Malassezia* species. Whether pre‐existing sensitization may predispose patients to TSW or whether TSW increases the sensitization risk would be best evaluated using longitudinal sampling, rather than our limited cross‐sectional data.

The study is limited by a small sample size, and thus, the results should be viewed as preliminary. Standard power calculations indicate that the minimal detectable difference with 14 patients with TSW and 10 comparators is 1.2‐fold the standard deviation; thus, any differences that were below that threshold may represent false negatives in our report. Yet overall, this research is the first to assess whether the sensitivities in patients with TSW are consistent with IgE biomarkers. The results are most notable for new‐onset reactivity to seafood and grains, in the setting of sensitization to dust mites, dander, and fungi. These data establish a hypothesis for future, longitudinal work in TSW‐related sensitizations.

## Author Contributions

A.V. ran the assay, wrote the manuscript, and performed analysis. N.S. helped write the paper and saw the patients. J.J. and P.T. assisted in writing the paper and performed some assays. I.A.M. oversaw the project, saw the patients, and wrote the paper.

## Disclosure

This research was supported by the Intramural Research Program of the National Institutes of Health (NIH). The contributions of the NIH author (s) were made as part of their official duties as NIH federal staff, are in compliance with agency policy requirements, and are considered works of the United States Government. However, the findings and conclusions presented in this paper are those of the author (s) and do not necessarily reflect the views of the NIH or the US Department of Health and Human Services.

## Conflicts of Interest

The authors declare no conflicts of interest.

## Supporting information


**Figure S1:** all70068‐sup‐0001‐Supinfo1.pdf.
**Figure S2:** all70068‐sup‐0001‐Supinfo1.pdf.

## Data Availability

The data that supports the findings of this study are available in the Supporting Information of this article.
