# Supplementary material for: Biomarkers Are Consistent With Patient‐Reported Allergic Sensitization in Topical Steroid Withdrawal
Source: Allergy. 2025 Sep 25;81(3):906–9. doi: 10.1111/all.70068 (PMC12954551; doi:10.1111/all.70068)
Supplement: Supplementary file 1 — Figure S1: all70068‐sup‐0001‐Supinfo1.pdf. Figure S2: all70068‐sup‐0001‐Supinfo1.pdf. [file ALL-81-906-s001.pdf]

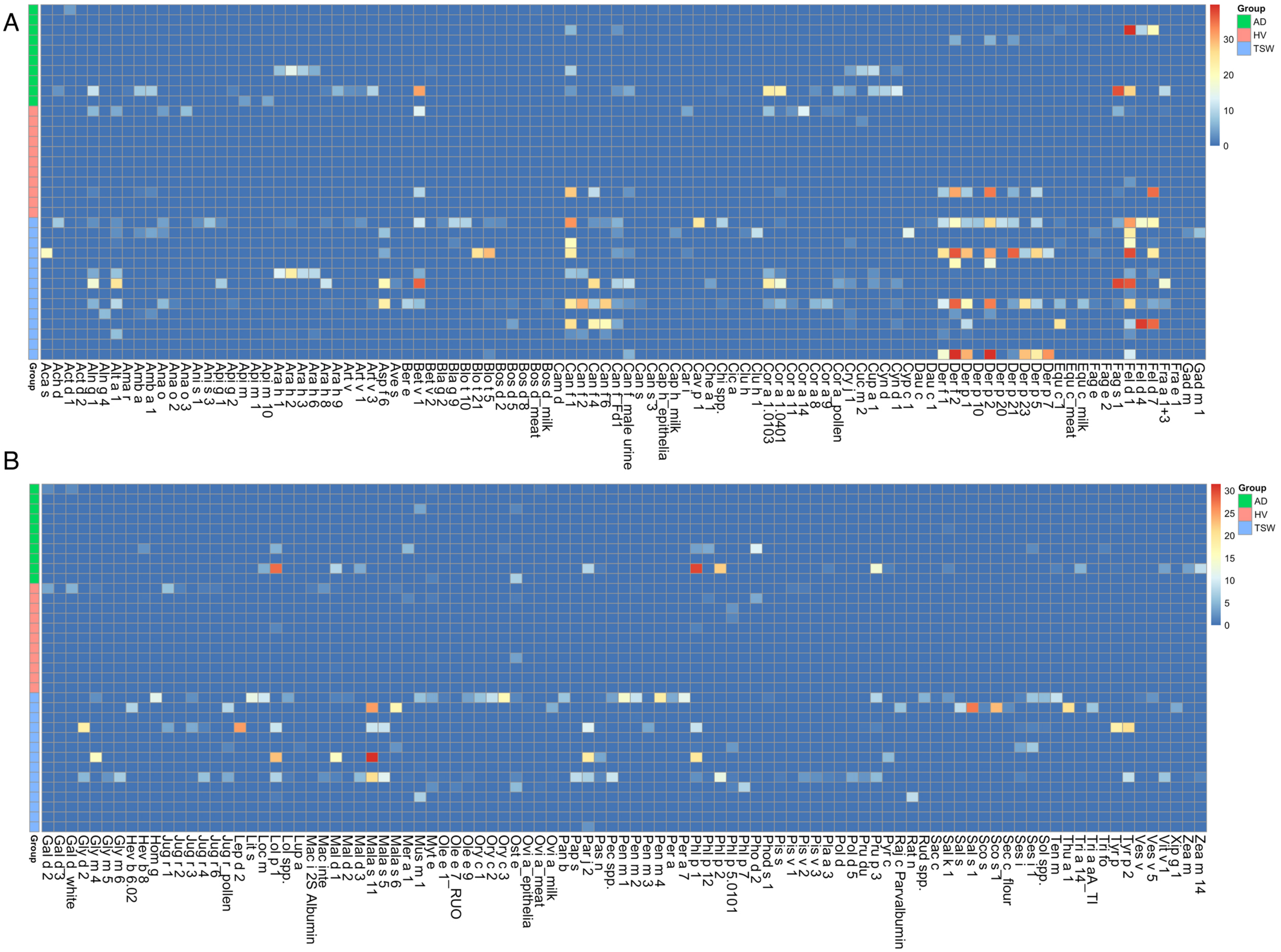

Supplemental Figure 1: Specific IgE reactivity between groups. (A-B) Specific IgE (kUA/L) for all assessed epitopes is displayed between TSW, AD, and HV cohorts.

Figure S1

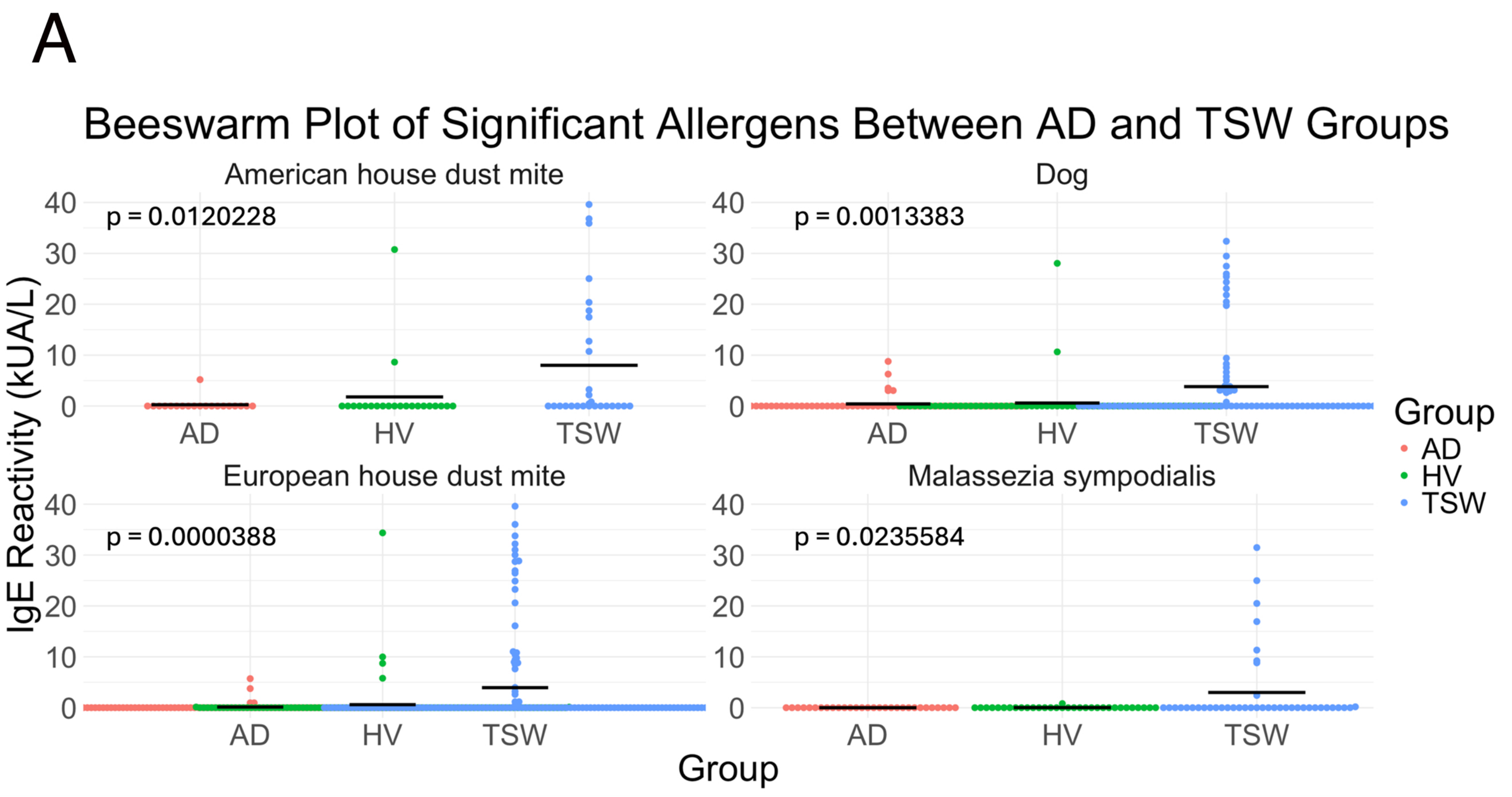

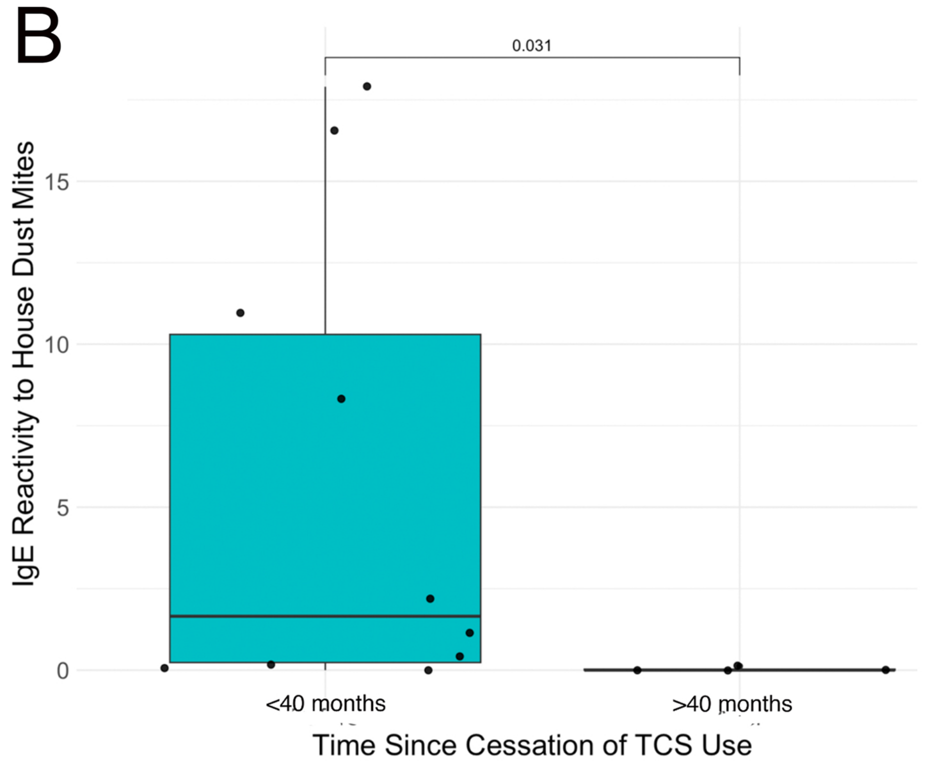

Supplemental Figure 2: Specific IgE reactivity to more specific categories of allergens. (A) Beeswarm plot of specific IgE (kUA/L) to significantly different allergens between TSW and AD cohorts determined by ANOVA with post-hoc Tukey HSD test. (B) Mean specific IgE (kUA/L) to house dust mite epitopes between patients with TCS avoidance times of less and greater than 40 months in a cohort of patients with TSW ($p = 0.031$, Student t-test)

Figure S2
